# Describing current use, barriers, and facilitators of patient portal messaging for research recruitment: Perspectives from study teams and patients at one institution

**DOI:** 10.1017/cts.2023.522

**Published:** 2023-04-05

**Authors:** Hailey N. Miller, Sierra Lindo, Laura J. Fish, Jamie Roberts, John Stover, Earl H. Schwark, Nicholas Eberlein, Dalia Mack, Margaret Falkovic, Christina Makarushka, Ranee Chatterjee

**Affiliations:** 1 School of Nursing, Johns Hopkins University, Baltimore, MD, USA; 2 Duke Cancer Institute, Behavioral Health and Survey Research Core, Duke University, Durham, NC, USA; 3 Department of Family Medicine and Community Health, Duke University, Durham, NC, USA; 4 Duke Clinical and Translational Science Institute, Recruitment Innovation Center, Duke University, Durham, NC, USA; 5 Duke Cancer Institute, Durham, NC, USA; 6 Duke University School of Medicine, Durham, NC 27710, USA

**Keywords:** Recruitment, patient portal, patient portal messaging, electronic health record, mixed methods, qualitative interviews

## Abstract

**Introduction:**

The electronic health record (EHR) and patient portal are used increasingly for clinical research, including patient portal recruitment messaging (PPRM). Use of PPRM has grown rapidly; however, best practices are still developing. In this study, we examined the use of PPRM at our institution and conducted qualitative interviews among study teams and patients to understand experiences and preferences for PPRM.

**Methods:**

We identified study teams that sent PPRMs and patients that received PPRMs in a 60-day period. We characterized these studies and patients, in addition to the patients’ interactions with the PPRMs (e.g., viewed, responded). From these groups, we recruited study team members and patients for semi-structured interviews. A pragmatic qualitative inquiry framework was used by interviewers. Interviews were audio-recorded and analyzed using a rapid qualitative analysis exploratory approach.

**Results:**

Across ten studies, 35,037 PPRMs were sent, 33% were viewed, and 17% were responded to. Interaction rates varied across demographic groups. Six study team members completed interviews and described PPRM as an efficient and helpful recruitment method. Twenty-eight patients completed interviews. They were supportive of receiving PPRMs, particularly when the PPRM was relevant to their health. Patients indicated that providing more information in the PPRM would be helpful, in addition to options to set personalized preferences.

**Conclusions:**

PPRM is an efficient recruitment method for study teams and is acceptable to patients. Engagement with PPRMs varies across demographic groups, which should be considered during recruitment planning. Additional research is needed to evaluate and implement recommended changes by study teams and patients.

## Introduction

Patient portals – secure, online platforms that are connected to the Electronic Health Record (EHR) – have been widely integrated into clinical practice across the United States of America (USA) since the enactment of the Health Information Technology for Clinical Health Act in 2009 [1]. Since this Act, their use by providers and patients has grown exponentially. In fact, nearly 40% of adults in the USA accessed their patient portal in 2020, a 13% increase from six years prior [2].

Patient portals are accessible via web browsers and mobile device applications and are used by patients for various functionalities related to their health and care delivery, including communicating with their healthcare providers, requesting medication refills, and viewing test results [2]. These convenient and accessible functions are associated with improved patient safety and satisfaction, enhanced patient-provider communication, and reduced patient uncertainty and anxiety [3]. As patient portal features have advanced, they are increasingly used for a host of other functions, including for interventions aimed at improving patient health behaviors and outcomes (e.g., medication adherence, preventative service use, and blood pressure) [4–7].

Given patient portal integration with the EHR and robust use among the US population, patient portals have started being leveraged for participant recruitment for clinical research studies [8–11]. Briefly, discrete data values in the EHR, such as diagnostic codes, lab values, and medication lists, are used to identify a target population. Following, recruitment invitations can be sent via the patient portal to the individuals identified in the EHR. This method, using the EHR cohort identification and patient portal recruitment messaging (PPRM), has been effective at identifying and enrolling individuals in clinical research studies and is an efficient recruitment method for some study teams [12–14]. Importantly, more than 90% of participants in a previous study agreed that PPRM for recruitment was an appropriate use of the patient portal [10]. Despite the rapid expansion of PPRM in the last five to seven years, the literature on acceptability and best practices for its use has not substantially grown, leaving limited guidance for institutions that offer or plan to offer PPRM as a recruitment method.

Identifying best practices and patient preferences for PPRM is critical to inform future expansion of PPRM as an effective recruitment methodology and to maintain and promote patient privacy and trust. Work in this area is scarce and, further, is often limited in scope to a single clinical trial or study. Additionally, to the best of our knowledge, no study to date has conducted qualitative interviews to assess patients’ perceptions and preferences regarding the use of the EHR and PPRM for research participant recruitment. To that end, at a single institution, we sought to understand the use of PPRMs used for research recruitment across several studies from both the perspectives of the study teams and patients.

We quantitatively describe the study teams that recruited via PPRM and the patient populations that were sent PPRMs for research recruitment during a defined period of time. Following, we conducted qualitative interviews among the study teams and patients to 1) understand study teams’ motivations, facilitators, and barriers to using PPRM as a recruitment method for research participant recruitment and 2) assess patients’ experience, perceptions, and preferences surrounding the utilization of the EHR patient portal for research recruitment purposes. We received Institutional Review Board (IRB) approval with waived consent in May 2021 as one of the Patients as Partners initiatives of the Duke Clinical and Translational Science Institute (CTSI).

## Materials and Methods

### MyChart for Recruitment Service Overview

MyChart is the patient portal associated with the Duke Health EHR “Maestro Care” (Epic Systems Corporation). Epic has a standard research recruitment request workflow that connects the patient with information about a research study and allows the patient to indicate interest (or non-interest) in learning more about the study. Within this standard workflow, there is a “Research” page in MyChart that stores the research invitations sent to the patient. The Duke Office of Clinical Research (DOCR) started using this workflow to send PPRMs to patients in October 2017. Since launching, through August 2022, PPRM has been used to support over 100 study teams and to send over one million PPRMs. When a study team is interested in sending PPRMs, they work with a DOCR Maestro Care analyst to create a computable phenotype, which is used to identify individuals in the EHR that meet basic eligibility criteria for the research study. Following this, the Recruitment Innovation Center provides support and templates to guide the study team in drafting their PPRM, including common language and formatting. They also review the message for readability and verify IRB approval before the DOCR analyst sends out the PPRMs. While the DOCR infrastructure is supported by the Duke School of Medicine and the Duke Clinical and Translational Science Award (CTSA), the individual study teams support the effort for the DOCR analyst to create the computable phenotype and send the PPRMs for their specific study. Currently, at this institution, the initial cost for the analyst’s effort of creating and testing the computable phenotype ranges, depending, in part, on the experience of the analyst, from $1,500 to $3,000, and the cost for sending PPRMs ranges from $150 to $350 per month.

**Fig. 1.**
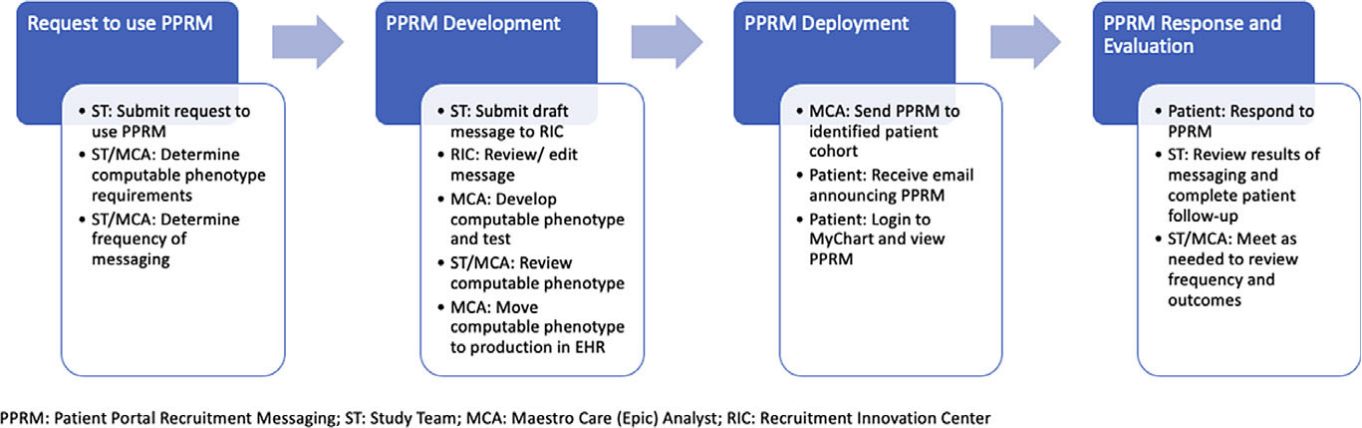
Processes for PPRM development and deployment at Duke health.

When a patient is sent a PPRM, they receive an email that notifies them that “MyChart has been updated with a research study you may be interested in viewing.” If a patient views the invitation, they have the option to respond to the invitation by hitting one of two buttons: “Interested” or “No, thank you,” which is captured in Maestro Care. The invitation may also include a website URL linking the patient to a study website, online pre-screener, or study consent form. If the patient selects “Interested,” a notification via Epic In Basket is sent to the respective study team. The notification provides information to screen the patient for eligibility and communicate with the patient about next steps. However, if a patient chooses to click the URL and does not click the “Interested” or “No, thank you” options, this is not tracked within Maestro Care, and the team is not sent a notification via Epic In Basket. This workflow is depicted in Fig. 1.

Prior to October 2017, to establish this workflow, DOCR engaged multiple stakeholders across the Health System to form an advisory group, which included individuals from the MaestroCare Optimization for Research program, the Duke IRB, and the Duke University Health System (DUHS) Compliance Office. The advisory group informed the development of processes and policies to protect patient privacy and support patient preferences. This included creating standard language on all PPRMs that provided an option for patients to “opt out” of receiving future PPRMs and establishing the processes to ensure that future computable phenotypes would exclude those individuals. The standard language also required that PPRMs indicate to patients that no individual had reviewed their health record – rather, a computer program had searched the EHR and identified them as someone who might be eligible for that particular study. These protections allowed the use of PPRMs without changes to the DUHS Notice of Privacy Practices (NPP), which had a “no cold-call” policy, and allowed a new method of direct-to-patient engagement of research participants. In March 2019, DUHS made changes to the NPP and the IRB “no cold-call” policy, allowing study teams more extensive options for direct-to-patient engagement across the health system, with appropriate safeguards and training requirements. All study teams planning to use direct-to-patient methods, including PPRMs, must undergo training on Duke’s new Recruitment and Engagement Policy and obtain IRB approval of their recruitment plans.

### Characterization of PPRM Utilization

To provide an overview of the utilization of PPRM at our Institution, we identified all research study teams that sent PPRMs and all patients that received a PPRM in a 60-day period between September 1, 2021, and October 29th, 2021 (inclusive). We excluded COVID-19-related PPRMs. This decision was made because during our 60-day period of interest, approximately 75% of all patients in the Duke Health System were sent at least one PPRM related to COVID-19 studies/programs. Following, we summarized these research studies’ characteristics and patients’ characteristics. We also summarized the message characteristics, including the number of PPRMs sent, viewed, and responded to by the patients.

### Participant Selection and Recruitment for Semi-structured Interviews

#### Study team members

Study teams were required to have sent PPRMs in the 60-day window stated above for a non-COVID study to be eligible to participate in the qualitative interviews. A survey was sent to all identified study teams, which collected basic information about their team’s experience using PPRM. The survey asked the study team to rate, on a scale of 1 (lowest) to 10 (highest), how useful PPRM was in helping them meet their recruitment goals; how useful PPRM was in comparison to other recruitment strategies; and if they would use PPRM again for this study population. Via email, we invited study teams that responded to the survey to participate in a semi-structured qualitative interview.

#### Patients

To be eligible to participate in the interviews, patients were required to be 18 years of age or older and a Duke Health patient with an active patient portal account, defined as logging in at least once during the last 12 months. To date, approximately 73% of all Duke Health patients have active accounts. They also were required to have received more than one PPRM message, one of which was required to be in the 60-day window stated above. We purposively sampled three groups of participants for the qualitative interviews: 1) individuals who viewed and responded to the PPRM (i.e., clicked “Interested” or “No, thank you”); 2) individuals who viewed the PPRM, but did not respond; and, 3) individuals who received a PPRM, but did not view the PPRM. We identified individuals eligible for this study using the EHR and the data request process described above. We aimed to have representation from all three groups and, within each group, diversity across race, gender, ethnicity, and age.

Traditional mail and phone calls were used to recruit participants in order to ensure we reached all three groups. Mailed letters included a request for patients to inform our team via email or phone within 10 days if they did not wish to be further contacted about the study or if they were interested in participating. If an opt-out or interested message was not received, patients were contacted via phone by study staff who reviewed the purpose and procedures of the study. If interested, verbal consent was obtained and a qualitative interview was scheduled. Recruitment began in October 2021 and concluded in May 2022.

#### Semi-structured interviews

We used a pragmatic qualitative inquiry framework for study team and patient interviews [15]. We developed semi-structured interview guides based on input from the project team. The project team included Duke CTSI Recruitment Innovation Center members, DOCR analysts, social and nursing scientists, and a primary care internist, all of whom have extensive experience using PPRMs to recruit patients for research. The interview questions for study teams focused on their general experience recruiting using PPRMs, including barriers and facilitators to using PPRM as a recruitment method, and suggestions for improving the process. The interview questions for patients focused on prior participation in clinical research, interaction with the PPRM, and suggestions for improving the process. The project team met biweekly throughout the interviewing process to discuss accrual progress and potential changes to recruitment and the interview questions. Interviews were conducted by three experienced qualitative interviewers between May 2021 and November 2022. All interviews were audio recorded.

### Data Analysis

Using descriptive statistics, we summarized responses to the study team survey. We also summarized the demographic characteristics of the patients who participated in the semi-structured interviews.

We used a rapid qualitative analysis exploratory approach to analyze the semi-structured interviews [16–18]. This approach results in a reduced timeframe and thus is more deductive and explanatory in contrast to traditional qualitative approaches [19]. The audio recordings of all interviews were summarized by the interviewers within 24 hours of being completed. We used a deductive template based on the interview guide to structure the analysis and to create a summary for each interview. The summary template included the main issues of interest based on the aims of the research. After developing the template, we tested it by having two team members code the same interview and compare and resolve discrepancies in findings. We used the final template to summarize all interviews. An experienced qualitative analyst conducted the first pass at summarizing the interview, and a second qualitative analyst reviewed the transcript and edited the coding template. Next, our team met to discuss and resolve discrepancies. Finally, we explored the summarized data with respect to the research objectives and produced a report of findings and recommendations.

## Results

### Characterization of PPRM Utilization

Thirty research study teams sent PPRMs in the 60-day window used for the current study, ten of which responded to our survey and were recruited to participate in the interviews (Table 1). Among the studies recruited to participate, seven were intervention studies, two were registries, and one was a cross-sectional survey. Across the ten studies, 35,037 messages were sent to patients who met the specified criteria in the 60-day period. The PPRMs were, on average, 245 words in length. In total, 33% of recipients viewed the message; 17% responded to a message; and 7% of recipients selected “Interested” upon receipt of the message. For patients that responded “Interested,” it took a median of two days to respond.

**Table 1.** Study and patient portal recruitment message characteristics

	Full sample	Qualitative sample
**Study characteristics**	*N* = 30	*N* = 10
Type of Study, *N* (%)		
Intervention	23 (77)	7 (70)
Registry	4 (13)	2 (20)
One-time Survey	3 (10)	1 (10)
Messages Sent, *N*	72,717	35,037
**Messaging characteristics**		
Word Count, *Mean*	258.8	245.4
Character Count, *Mean*	1380.7	1380.5
Link Provided, N (%) Yes	13 (43)	6 (54)
Viewed, *N* (%)	23,495 (32)	11,601 (33)
Responded, *N* (%)		
Interested	5,101 (7)	2,444 (7)
Declined	6,909 (10)	3,439 (10)
Days to response, *Median*	2.2	2.2
Days to response if interested, *Median*	1.1	2.0

Among patients who received PPRMs from the ten studies described above, a slight majority (52.1%) were male. Patients ranged in age from 0 to 100 years. Additionally, the majority of patients were identified as White race (60.9%), followed by 34.8% who were identified as Black or African American race and 3.8% as Asian race. A small proportion of patients were identified as American Indian or Alaskan Native race (0.6%) or Native Hawaiian or Pacific Islander (0.1%), and 3.8% were identified as being of Hispanic or Latino ethnicity (Table 2). Across all sociodemographic groups, more than 20% of recipients viewed the PPRM, except for individuals 18–25 years of age, who viewed the PPRM 16.7% of the time. Individuals who were 65–74 years of age viewed the PPRMs more frequently than any other sociodemographic group, with 39.2% of patients viewing the PPRM. Among individuals who responded, females, individuals who were identified as not being of Hispanic or Latino ethnicity, and individuals 65–74 years old responded “Interested” at higher rates compared to males, individuals who were identified as being of Hispanic or Latino ethnicity, and other age groups (Table 2). Individuals whose race was identified as American Indian and Alaskan Native also responded “Interested” more frequently than other racial groups (11.1%); however, the overall number of individuals sent a message in this group was small (*n* = 207), so it is difficult to make comparisons.

**Table 2.** Patient interaction with patient portal recruitment messages by sociodemographic characteristics

Sociodemographic Characteristics	Messages Sent, N (%)	Interaction with PPRM*		
		Viewed, N (%)	Interested, N (%)	Declined, N (%)
Total	35,037	11,601 (33)	2444 (7)	3439 (9.8)
Sex				
Female	16780 (47.9)	5819 (34.7)	1300 (7.7)	1639 (9.8)
Male	18257 (52.1)	5782 (31.7)	1144 (6.3)	1800 (9.9)
Race				
Black or African American	12188 (34.8)	3234 (26.5)	740 (6.1)	774 (6.4)
Asian	1332 (3.8)	339 (25.5)	41 (3.1)	96 (7.2)
White	19715 (56.3)	15981(38.2)	1563 (7.9)	2428 (12.3)
American Indian or Alaskan Native	207 (0.6)	73 (35.3)	23 (11.1)	20 (9.7)
Native Hawaiian or Pacific Islander	41 (0.1)	10 (24.4)	2 (4.9)	5 (12.2)
Not Reported or Declined	811 (2.3)	218 (26.9)	41 (5.1)	70 (8.6)
Other	744 (2.1)	449 (25.8)	90 (4.7)	103 (6.2)
Ethnicity				
Hispanic or Latino	1331 (3.8)	370 (27.8)	76 (5.7)	104 (7.8)
Not Hispanic or Latino	32474 (92.7)	10834 (33.4)	2290 (7.1)	3216 (9.9)
Not Reported or Declined	1232 (3.5)	397 (32.2)	78 (6.3)	119 (9.7)
Age				
0-17	1633 (4.4)	448 (27.4)	108 (6.6)	156 (9.6)
18-24	2383 (6.8)	398 (16.7)	55 (2.3)	101 (4.2)
25-34	1756 (5.0)	569 (32.4)	93 (5.3)	150 (8.5)
35-44	2517 (7.2)	795 (31.5)	153 (6.1)	203 (8.1)
45-54	4382 (12.5)	1394 (31.8)	303 (6.9)	403 (9.2)
55-64	6850 (19.6)	2382 (34.8)	518 (7.6)	705 (10.3)
65-74	9828 (28.1)	3848 (39.2)	836 (8.5)	1172 (11.9)
75-84	4666 (13.3)	1550 (33.2)	338 (7.2)	479 (10.3)
85 and older	1022 (2.9)	217 (21.2)	40 (3.9)	70 (6.8)

Notes: PPRM, patient portal recruitment message.

* The percentages displayed in this column are reflective of the % that responded among that specific demographic group or row [i.e., total number of females responded / total number of females messaged].

### Study Team Members

#### Survey responses

Survey responses by study team members are described in Table 3. Half of the study teams rated PPRM with a “10” on usefulness in meeting recruitment goals. Seven teams responded that, in comparison to other strategies, PPRM was more useful in meeting recruitment goals, while three teams said it was the same as other strategies. All but one team responded that they would use PPRM again for the same population.

**Table 3.** Study team survey responses

Prompt	Response, *N* (%)
	***N* = 10**
**On a scale from 1 (lowest)-10 (highest), how useful is/was MCRM in helping you to meet your recruitment goals for this project?**	
6	1 (19)
7	2 (20)
8	1 (10)
9	1 (10)
10	5 (50)
**In comparison to your other recruitment strategies, how useful is/was MCRM in helping you meet recruitment goals for this project?**	
Less useful	0 (0)
Same	3 (30)
More useful	7 (64)
**Would you use MCRM again for this study population?**	
No	0 (0)
Maybe	1 (10)
Yes	9 (90)

#### Study characteristics

Of the ten studies of interest, we completed interviews with six study teams. Five study teams were recruiting participants locally, and one was a nationwide study. Three were pediatric studies, one focused on diet in adults, one study tested a decision support tool for early breast cancer treatment decisions, and one focused on diabetes in adults.

#### Use of PPRM for recruitment

Three of the six studies used PPRM as a recruitment method from study conception, and three incorporated PPRM after experiencing problems with their original recruitment plans. Some opted to link potential participants directly to study websites/e-consent, while others used the In basket function described above. One study that used the In basket function noted there were extra steps to follow up with people who were interested; however, this feature fit their overall study design.

#### Experience using PPRM for recruitment

Study teams reported that PPRM recruitment allowed for more efficient, targeted recruitment and were enthusiastic about the amount of time this recruitment method saved. They did note some difficulties with the process of setting up PPRMs and an overall low response rate, with one team estimating a response rate of 2%–5%. This was verified with numbers reported by other study teams. Study teams shared the sentiment that the increased efficiency of recruitment outweighed the cost of using this recruitment method. One study team specifically noted that the ability to modify the criteria for patient selection over time was useful in meeting their recruitment goals. Study teams also provided suggestions for improving patient portal recruitment, described in Table 4.

**Table 4.** Study team recommendations for improving patient portal recruitment messaging

Recommendation	Preferences
Provide training to study team	Provide a one-page document that explains the back end of the patient portal and EHR. This will help study staff understand the methodology and articulate it on study-related materials, including dissemination.
Simplify intake process	Simplify intake form and process for setting up selection of patients in the EHR.
Link Epic to REDCap	Provide the option to bypass the “interested” button and automatically import information from EHR to REDCap to facilitate tracking and outreach.
Streamline study information to patients	Simplify the steps patients need to take to get to the information about the research study.
Develop system for tracking efficacy	Track the number of messages sent and those that lead to enrollment. Provide a report template that study teams can use to track recruitment outcomes.
Provide additional response options for message	Add other options for patients to click on within PPRM such as interested/not interested/learn more

### Patients

#### Demographics

A total of 28 patients completed an interview. Among these participants, six did not view the message; nine viewed the message but did not respond; and thirteen viewed the message and responded. The majority of participants identified as female (*n* = 17) and of White race (*n* = 16), followed by eight respondents who identified as being of Black or African American race, and three who identified as being of Asian race. Three participants identified as being of Hispanic or Latino ethnicity. The mean age of these patients was 49 years old.

#### Experience with the patient portal

Participants reported a wide range in the frequency they used the patient portal, with some as often as weekly. Their use was commonly related to the participant’s specific health context, such as having a chronic disease that necessitated frequent appointments or interactions with the health system. Most participants reported using a combination of the website and smartphone application (app) to access their patient portal account. The participants reported using several patient portal functions that align with previous literature [2].

#### PPRM recall, response, and experience with research

Across all of the participants interviewed, roughly half recalled receiving a PPRM about a research study and more than half reported prior experience participating in research studies. Patients were generally supportive of receiving PPRMs for research recruitment purposes; however, many noted difficulty in navigating to the PPRM. Surprisingly, the group with the most consistent PPRM recall was the one of participants who viewed but did not respond (*n* = 9). Many patients who viewed but did not respond reported current or recent participation in other research studies as a reason for not responding. Among participants who responded to the PPRM, more than half reported no previous research participation before receiving the PPRM. Lastly, most participants who did not open the research message (*n* = 6) reported no previous participation in research. However, all but one of these participants specifically stated that they were generally interested in participating in research.

It is important to note several participants mentioned reluctance to participate in research during the period being studied due to the COVID-19 pandemic, and they were not interested in studies that required in-person contact.

#### PPRM content and frequency

Generally, participants preferred receiving a PPRM with a subject line specific to the research study being conducted rather than a generic subject line (e.g., there is a research study that may be of interest). When discussing what information they use to decide to participate in a research study, participants discussed study requirements (e.g., blood draw, in-person visits) and why the research topic being studied is relevant to them. Participants indicated a reminder message would be acceptable, particularly if they had not viewed the message yet. Preferences on frequencies of reminders varied across participants, from 2 to 3 times per month to every other month. Participants consistently expressed that they are most interested in receiving PPRMs directly relevant to their health.

#### Suggestions for improving PPRM and research recruitment

Participants had constructive feedback regarding what contributed to their lack of response and suggestions for improving PPRMs, detailed in Table 5. These suggestions include improvements for the initial message that patients receive, such as what information to include, and sending reminder messages. Additionally, they cited a need for improvements in the patient portal to be able to navigate to the PPRMs more easily. As an alternative to PPRM, participants identified direct contact by phone or email as an effective way to get their attention regarding potential research studies, in addition to standard methods of referral to research studies, such as provider recommendations and letters. A few patients suggested text messaging as an acceptable method for introducing patients to potential research studies. Across all groups, there was confusion about the differences between email messages and PPRM.

**Table 5.** Patient feedback and suggestions for improving patient portal recruitment messaging

Complaint	Context	Recommendation	Preferences	Relevant Group		
				Did not open	Viewed, no response	Responded
Meaning of research message was unclear	Participants were concerned if message referred to risk of a serious health issue/ were unsure if the message indicated bad news.	Emphasize personal relevance	Confirm that the message states the relevance to an individual’s health conditions	X	X	X
Message did not include enough information	Participants reported that the initial message did not contain enough information to entice them to express interest.	Provide study details in initial message	Include as much information as possible about the research study in the email. Suggestions: Title Overview of research aims Study website Eligibility criteria Time commitment Study procedures Compensation In-person or virtual Contact information	X	X	X
Inability to set preferences	Participants reported that they preferred being contacted for different types of studies and at varying frequencies.	Provide patients with ability to set preferences for research	Allow patients to limit the number and type of studies they are contacted by/for.		X	X
Unable to locate the message after initial viewing	Participants logged into the portal to complete a healthcare-related task and forgot to follow up once they finished. Participants noted they were unable to find the message the next time they logged into the patient portal.	1. Make research tab more prominent	The research tab should be easy to find in the patient portal system.	X	X	X
		2. Send reminder messages	Send reminders (1-3) if the patient does not respond.		X	X
Experienced technical difficulties	Participants who used a smartphone app reported responding multiple times, but the link connected the participant to a webpage that was not compatible with their smartphone.	Optimize use on the smartphone	Allow patients to complete steps within the smartphone app.		X	X

## Discussion

Patient portal recruitment messaging (PPRM) as a recruitment method for clinical research studies has rapidly gained popularity in the last several years. Our study uncovers important insights on current PPRM utilization and areas of improvement to enhance the study team and patient experience. Overall, study teams and patients reported positive experiences and perceptions about PPRM, in addition to its future use for research recruitment. However, study teams and patients alike shared areas of criticism and opportunities for growth for this evolving technology and recruitment method, some of which are already being responded to at our institution.

Feedback from study teams revealed that PPRM was an efficient recruitment method; it required minimal effort from the study staff and facilitated reaching a large number of people. Study team members noted that optimizing the onboarding (i.e., initiating PPRM recruitment) and tracking processes would improve the overall experience. In response, the DOCR Maestro Care team is developing a short training video that will contain an overview of PPRM processes. Additionally, it was recommended that teams have the option to import information from the EHR directly into a database management software, such as REDCap [20]. This workflow would be particularly relevant for teams that use the EHR for screening. This workflow is possible at our institution; however, this is not a function of PPRM, and it requires additional IRB approvals (e.g., HIPAA waivers). In the future, our institution is aiming for these processes to be completed within the EHR so that data does not have to be stored elsewhere.

An area of criticism by patients was that there was not enough information in the PPRM to motivate them to learn more about the study or express interest. Patients advised our team to include as much information as possible in the PPRM, and, in particular, information about how the study pertains to the patient’s health. Although the literature is limited on best practices for PPRM message content, this finding was supported in Plante *et al*, a study evaluating recruitment yields between two PPRM lengths, a short and a long version, among older adults [21]. Results of this study indicated that a longer PPRM with more study-related content improved response rates from potential participants.

Patients indicated that they would prefer the ability to set individual preferences on how often to be contacted and for which type of topics or studies to be contacted about. This may be particularly important to consider as it was evident within our sample that the preferred frequency of messaging varied. A previous study conducted by Samuels *et al*. surveyed participant opinions one month after recruitment using a patient portal and found that participants noted it would be acceptable to be contacted as often as studies came up [13]. To date, no customization for contact preferences has been implemented at our Institution beyond the ability to opt out of messaging. This is largely because patient preference data can be difficult to utilize and can lead to exclusion from future studies. For example, if a patient chooses to opt out of studies that involve a treatment intervention, but later develops an illness, they would not receive an invitation to participate in novel drug or treatment trials. Before implementing this recommendation, consideration must be given to if and how these preferences would evolve with major life events and medical diagnoses.

Another theme that emerged was that patients had difficulty navigating to the PPRM after receiving an email notification. This concern regarding usability aligns with previous literature, which has indicated that navigating the patient portal is a barrier to its use. [22] For example, in a systematic review of patients’ attitudes toward using the patient portal for chronic disease management, 41% of articles reported user-friendliness of the portal as a barrier [23]. To alleviate patients’ frustrations with navigating to the message, it might be beneficial to include basic instructions in the email to help them navigate to the “Research” page, which our institution has, or include a shortcut button on the menu bar of the patient portal.

In addition to receiving PPRMs, patients indicated other modalities would be appropriate to use for research invitations, including phone, email, text, and postal mail. They felt that these alternative outreach modalities could be used simultaneously to PPRM. Previous studies have found success in using several direct-to-patient outreach methods and have demonstrated improved recruitment yields when using a multi-modal approach [24]. For example, researchers at Johns Hopkins University used text, email, and PPRM to recruit individuals to join a COVID-19 Registry, finding email to be the most effective [25]. A second study comparing four different recruitment methods found that PPRMs were the most effective approach; however, using clinic recruitment, letters, and emails allowed the study team to reach a wider net of patients, largely contributing to their recruitment success [26]. In that study, letters reached a larger proportion of Black adults, a finding found in previous studies as well [27].

Our utilization data demonstrated that interaction with PPRMs varied by demographic groups. For example, young adults (ages 18–25) viewed and responded to PPRMs at much lower rates than any other demographic group. This information could be used to inform which types of studies would benefit most from adding PPRM to their recruitment plan, in addition to which populations likely require alternative recruitment methods to increase their participation in research. Additional research is needed, however, to establish the effectiveness of recruitment modalities in diverse populations.

Despite the known weaknesses in PPRMs with regard to user demographics, a benefit of PPRM uncovered by study team members was the ability to target specific subgroups in recruitment [28]. For example, a study team can request to only send PPRMs to patients in demographic groups that are traditionally underrepresented in research, rather than to any person that meets the computable phenotype criteria. Similarly, this can be modified throughout the recruitment period and, therefore, adaptive to the study’s recruitment progress and goals. This is a function that many traditional and non-digital recruitment methods cannot offer. We encourage institutions offering direct-to-patient recruitment to promote this functionality to study teams to reduce the occurrence of biased and non-representative participant samples.

This study had limitations. First, we only summarized utilization data over a 60-day period. However, there is no reason to believe that this period differs from other periods in a meaningful way, except for the conduct of COVID-related studies which we excluded from our sampling frame. Second, several studies included a link to an outside form for the recipient to learn more, express interest, or provide consent. For this reason, our response rates for these studies, particularly for individuals who were “Interested,” are likely underestimated. Third, it should be noted that the utilization data was summarized and the interviews were conducted during the COVID-19 pandemic, which may have influenced the results. Lastly, this is a qualitative study; therefore, our findings represent the subjective perception of the study participants. For the qualitative portion of the study, we used purposive sampling to explore a range of perspectives of patients and study teams, which may not align with objective measures of acceptability of PPRM use for research recruitment. Additional research, with larger patient samples and a diversity of study types, is needed to capture more nuanced perspectives and objective measures of utility related to PPRM recruitment.

Despite these limitations, the study has many strengths. We report data characterizing PPRM usage among several types of research study teams and various patient populations. We also report qualitative data on both study teams’ and patients’ perceptions and preferences for PPRM, adding to the limited knowledge of best practices for this relatively new direct-to-patient recruitment methodology. Moreover, our sampling strategy for interviews aimed to achieve diverse representation and included patients who did not view or respond to the PPRM.

## Conclusion

Our findings underscore the usefulness of PPRM as a recruitment tool for study teams and demonstrate that PPRM can be used to support recruitment across various populations and study types. Our findings also highlight that patients are supportive of using PPRM for research recruitment and are comfortable being contacted by this method. Based on patient interviews, several changes can be made to the current PPRM methodology to improve the experience for potential research participants, including supporting the customization of contact preferences for PPRM outreach, providing more detailed study information, and refining the technology for the end-user. As changes are made to improve PPRM methodology, it is important that institutions and researchers evaluate the effectiveness of PPRMs in supporting engagement and participation in clinical research and, further, evaluate the effectiveness among underrepresented populations in research.
